# Gingival Reactive Lesions in Orally Rehabilitated Patients by Free Revascularized Flap

**DOI:** 10.1155/2018/2474706

**Published:** 2018-08-12

**Authors:** Gianluca Tenore, Ahmed Mohsen, Giorgio Pompa, Edoardo Brauner, Andrea Cassoni, Valentino Valentini, Antonella Polimeni, Umberto Romeo

**Affiliations:** Department of Oral and Maxillofacial Sciences, “Sapienza” University of Rome, Rome, Italy

## Abstract

The aim is to discuss four cases of gingival reactive hyperplastic lesions in patients with a history of excision of oral neoplastic lesions and rehabilitation by a free revascularized flap of the iliac crest. One female and 3 male patients were referred due to the presence of exophytic lesions at the rehabilitated sites. The clinical examination revealed that the poor oral hygiene was the common trigger factor in all the cases, in addition to trauma from the upper left second molar in the first case, pericoronitis related to a partially erupted lower right third molar in the third case, and poor stability of an upper removable partial denture in the fourth case. All the cases were subjected to elimination of these suspected triggering factors, exclusion of dysplasia, excisional biopsy by CO2 laser, and five follow-up visits. The histological examination of all the cases confirmed the diagnosis of pyogenic granuloma. These presented cases suggest that the limitations in oral functions and maintaining the oral hygiene measures following the free revascularized flap reconstruction surgery probably played a role in the development of gingival reactive hyperplastic lesions with presence of trigger factors such as local trauma, chronic infection, or inadequate prosthesis.

## 1. Introduction

Reactive hyperplastic lesion is defined as an excessive proliferation of connective tissue in response to chronic irritation. In the oral cavity, the gingival reactive hyperplastic lesions are pyogenic granuloma (PG), peripheral fibroma, fibroepithelial hyperplasia, peripheral ossifying fibroma, and peripheral giant-cell granuloma. The gingival reactive hyperplastic lesions are commonly described as “epulides,” which is a Greek word, means “on the gingiva.” However, this commonly used term is only describing lesions clinically present on the gingiva without specifying the nature of the lesion [1, 2].

PG was firstly described in 1897 by Poncet and Dor [3]. It is defined to be an inflammatory hyperplasia that usually exists in response to local low-grade irritants, traumatic injury, hormonal change, or certain medications. Focal epithelial hyperplasia is preferred instead of its classic term “PG” because the lesion is not precisely a granuloma or an infection [1, 4].

PG has a more female incidence than male with a ratio of 3 : 2. The most common site is the keratinized gingiva in about 75% of cases, followed, respectively, by tongue, lips, and buccal mucosa. It occurs more commonly in the maxilla than in the mandible and in the anterior region than the posterior [4]. Presence of periodontal disease and dental calculus are frequently suspected as initiating causative factors. In the last few years, PG and peripheral giant-cell granuloma have been considered the most common reactive lesions that appeared in association with implants. Elimination of triggering factors and surgical removal are recommended in the management protocol [1, 2].

The reconstruction of bone continuity defects and solving some complications such as facial contour disfigurement by a free revascularized flap (FRF) have become a valuable part in the management of head and neck cancers. The idea of FRF is the transplantation of bone segment with muscle and skin allowing a simultaneous reconstruction of hard and soft tissues [5].

The high success rate of FRF reconstruction is about 95–97% due to the refinement of microvascular and magnification instruments; however, rare complications still occur. The complications are divided into recipient site and donor site complications. The most common complications of recipient site are related to vascular thrombosis [6].

Many cases of hyperplastic/inflammatory response and formation of granulation tissues around implant abutments in the orally rehabilitated sites by FRF are reported. Some authors speculate that the unsuitability of skin tissues around implants and the negative reaction of the skin in the oral cavity may be the reason of this complication [5].

Presented here are four cases of gingival reactive lesions (mainly PG) in patients with a history of excision of oral neoplastic lesions and rehabilitation by a FRF of the iliac crest.

## 2. Materials and Methods

The cases are 1 female and 3 males. The management was started with full clinical examination consisting of medical, dental history, and radiographic investigation.

Suspected triggering factors were diagnosed and eliminated for each patient. Conventional blade incisional biopsy was performed after two weeks of the first visit. All the collected biopsy samples were fixed in a 10% neutral buffered formalin solution. The histopathological evaluation confirmed the absence of dysplasia in all the samples.

All the patients were informed about the advantages and disadvantages of laser surgery, signed an informed consent, and managed by the same surgeon.

Excisional biopsies were performed with local anesthesia (mepivacaine) by carbon dioxide (CO_2_) laser (wavelength of 10,600 nm; model SMART US-20D, DEKA®, Florence, Italy), with these parameters: power of 1.5–1.7 watts on pulsed wave (PW), frequency of 100 Hz, (power density 1432.12 W/cm^2^, energy density 14.32 J/cm^2^), and spot diameter of 400 *μ*m [7].

A 0.2% chlorhexidine spray (Corsodyl spray, GlaxoSmithKline Consumer Healthcare S.p.A., Baranzate, Milan, Italy) and a 0.5 ml of amino acids and sodium hyaluronate gel (Aminogam gel, Errekappa Euroterapici Spa, Milan, Italy) were prescribed three times daily for one week. The patients were informed to eat cold and soft food and to avoid hot and spicy food on the day of the intervention.

Five follow-up visits were performed according to this schedule: one week, three weeks, three months, six months, and one year after the surgery, in order to evaluate the healing process and the possibility of recurrence.

## 3. Case Series

### 3.1. Case I

**Figure 1:**
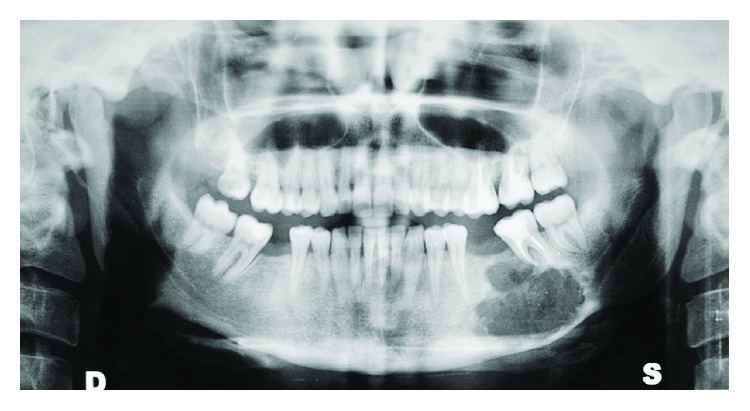
Panoramic X-ray before the excision of ossifying fibroma.

**Figure 2:**
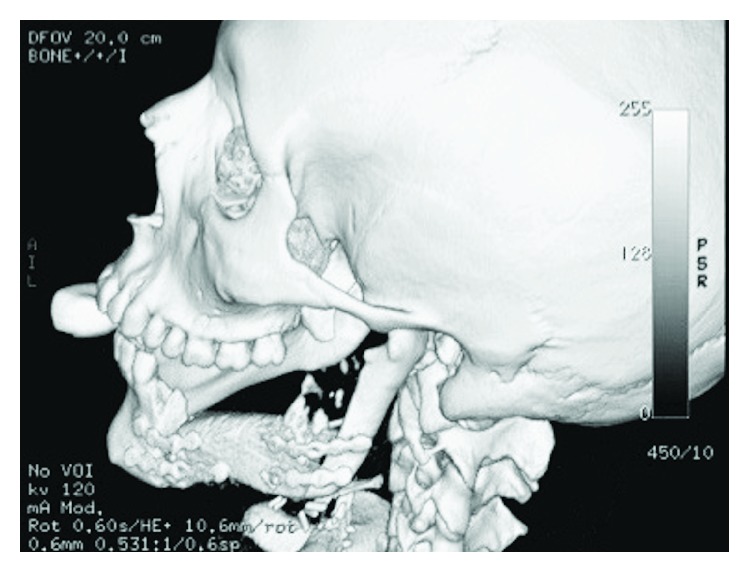
Radiographic image after reconstruction by FRF.

A 28-year-old female complained of an intraoral swelling in the lower left region. This swelling appeared few months ago. There was a complaint of bleeding on brushing without pain. Regarding her medical and dental history, she was suffering from ossifying fibroma at the left premolar-molar region of the mandible (Figure 1). It was excised and simultaneously rehabilitated by a FRF of iliac crest in 2013 (Figure 2).

**Figure 3:**
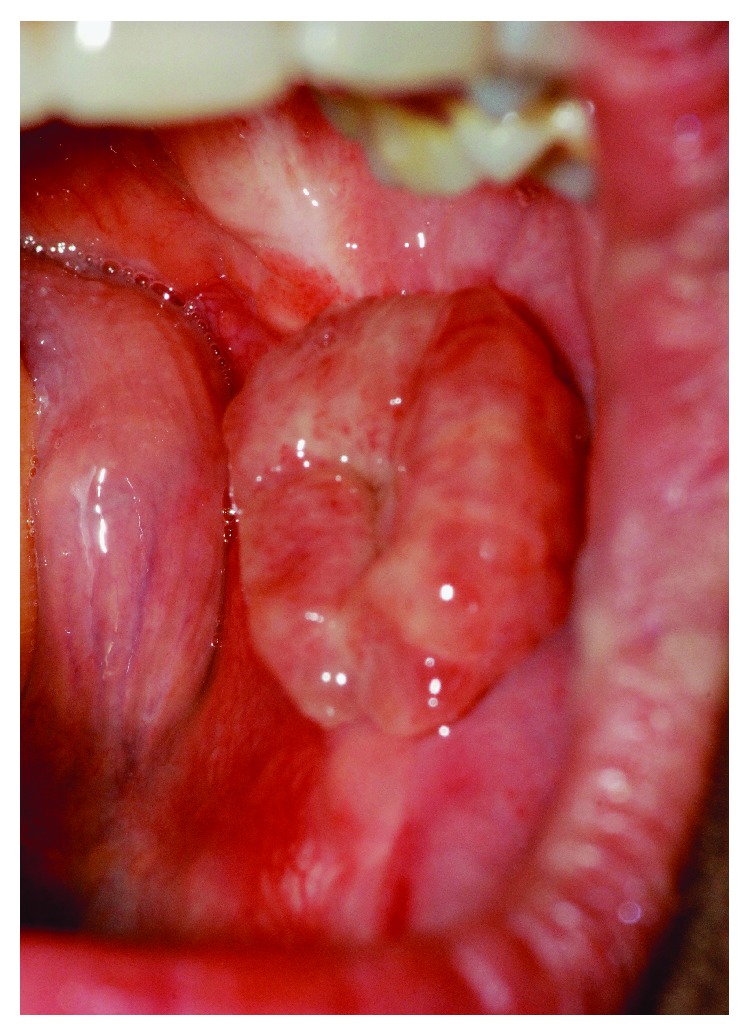
Clinical preoperative.

The oral examination revealed that an erythematous exophytic sessile lesion with granulomatous appearance and soft-elastic consistency on the lower left retromolar region. This lesion developed after approximately 2 years of the reconstruction by FRF (Figure 3).

A presence of mechanical irritation at the lesion area related to the upper second left molar was observed. Radiographic investigation did not show any bone resorption in relation to the lesion.

The provisional diagnosis was probably a reactive lesion like PG or peripheral giant-cell granuloma. Routine blood tests, exclusion of dysplasia by cold-blade incisional biopsy, and elimination of contributing triggering factors were done. Smoothing of the cusp tips of the upper left second molar was done in addition to improvement of the oral hygiene.

**Figure 4:**
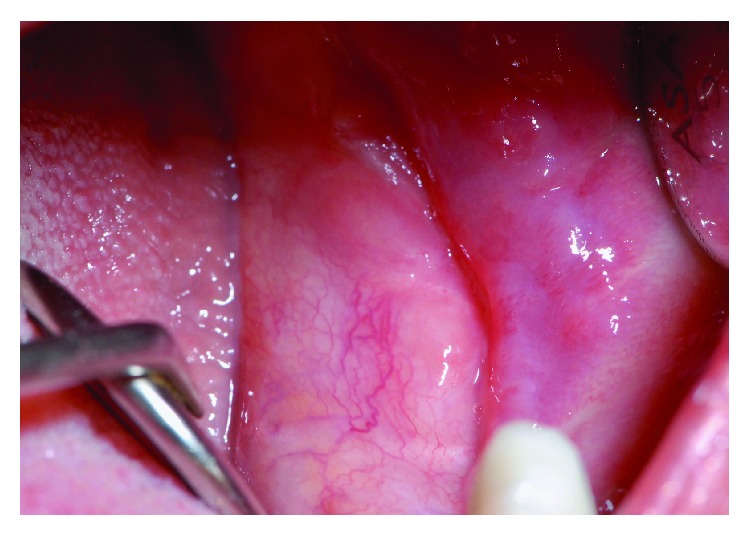
One-year follow-up.

Complete excision of the lesion by CO_2_ laser was performed under local anesthesia with the help of Allis forceps. The histological examination of the excised lesion confirmed the diagnosis of PG (Figure 4).

### 3.2. Case II

A 58-year-old male had a history of ameloblastoma at the right side of the body of the mandible. Excision and hemimandibulectomy were performed in 2011 with simultaneous reconstruction by a FRF of the iliac crest. It was rehabilitated with five prosthetic implants eight months later. He came for consultation of an intraoral swelling in the lower right area that appeared a few months ago after about 3 years of the reconstruction by FRF.

The oral examination showed an exophytic lesion, mostly sessile with granulomatous appearance and soft-elastic consistency related to the implants in the right incisors bicuspids region, from the lower right central incisor region to the first molar region on the same side. The radiographic investigation did not show any bone resorption in relation to the lesion around the implants.

Routine blood tests, exclusion of dysplasia by cold-blade incisional biopsy, and elimination of contributing triggering factors were performed. It was suggested that the triggering factor was the poor oral hygiene, thus the prosthetic crowns and bridge were removed for three weeks to facilitate the control of bacterial infection and to promote better tissue regeneration. Complete excision of the lesion by CO_2_ laser was performed under local anesthesia with the help of suture 3-0.

Another surgical intervention was performed with CO_2_ laser for recontouring the gingiva around the implants and to facilitate the cementing of implant prosthesis.

In the three-month follow-up visit, a recurrence was observed. A further intervention was performed by CO_2_ laser, with motivating the patient on the importance of maintaining the oral hygiene measures in order to ascertain the complete elimination of triggering factors. The histological examination confirmed the diagnosis of PG.

### 3.3. Case III

**Figure 5:**
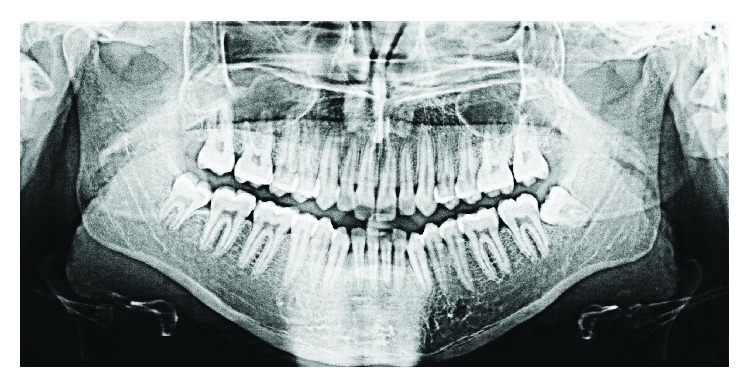
Panoramic X-ray before the excision of mucoepidermoid carcinoma.

**Figure 6:**
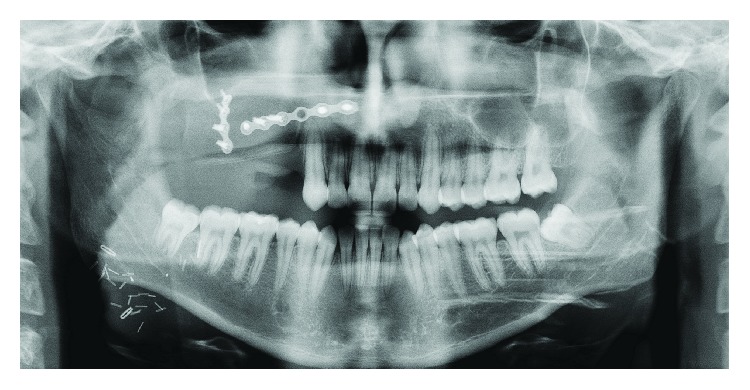
Panoramic X-ray after reconstruction by FRF.

A 19-year-old male was referred for consultation of a painless mass in the right retromolar area that developed few weeks ago. The medical and dental history revealed that in 2015 an excision of moderately differentiated mucoepidermoid carcinoma at the upper right posterior molar region and hemimaxillectomy were carried out with simultaneous reconstruction by a FRF of the iliac crest (Figures 5 and 6).

**Figure 7:**
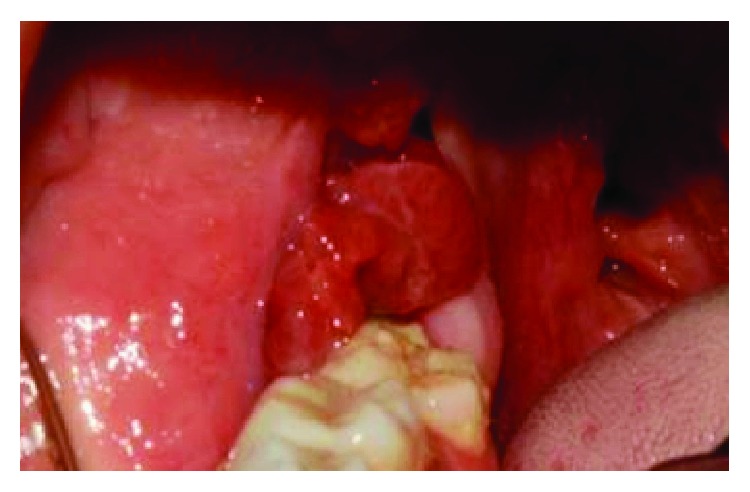
Clinical preoperative.

The oral examination revealed an exophytic, mostly pedunculated lesion, with irregular granulomatous appearance and elastic consistency on the lower right retromolar area related to a partially erupted lower right third molar (Figure 7). The radiographic investigation did not show any bone resorption at the site of the lesion.

Routine blood tests, exclusion of dysplasia by cold-blade incisional biopsy, and elimination of contributing triggering factors were performed. It was decided to excise the lesion by CO_2_ laser under local anesthesia and to extract the lower right third molar which might be the cause of chronic irritation.

**Figure 8:**
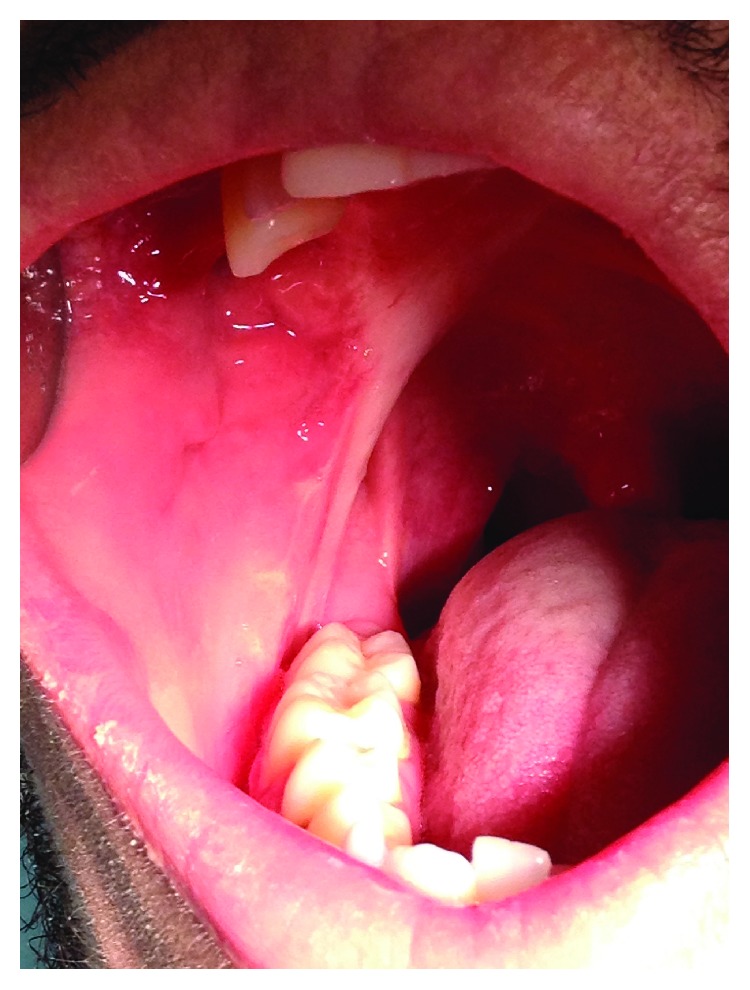
One-year follow-up.

The histological examination revealed a benign lesion with vascular structures and diffuse inflammatory infiltrate of granulocytes and neutrophils (Figure 8).

### 3.4. Case IV

A 21-year-old male was examined in our outpatient clinic complaining of a painless swelling in the upper left posterior region. Regarding his medical and dental history, left hemimaxillectomy, adenoidectomy, and partial removal of zygoma were carried out in 2001 due to a rhabdomyosarcoma in the left maxillary sinus. It was simultaneously reconstructed by a FRF of iliac crest, followed by radiotherapy and chemotherapy before and after the surgical intervention.

The oral examination showed exophytic, mostly pedunculated lesion with irregular granulomatous appearance and elastic consistency on the upper left posterior region related to the buccal flange and the fitting surface of the upper removable partial denture (RPD). The radiographic investigation did not show any bone resorption at the site of the lesion.

Contributing triggering factor was the poor stability of RPD. It was decided not to wear the RPD for two weeks. Routine blood tests, exclusion of dysplasia by cold-blade incisional biopsy, and the excision of the lesion by CO_2_ laser under local anesthesia were performed. The histological examination revealed a benign lesion with vascular structures and diffuse inflammatory infiltrate of granulocytes and neutrophils, in addition to focal aspects of abscess formation.

Deepening of the buccal vestibule by CO_2_ laser after the three-week follow-up has been done responding to a request from the prosthodontic department, to remake the RPD with better stability.

## 4. Discussion

The management of head and neck cancer has been improved by the introduction of microvascular surgery and FRF reconstruction. The ability of tissue transfer from a distant site enables the surgeons to reconstruct the bone and the soft tissues in a single-staged procedure [8]. The most common donor sites for the reconstruction of maxilla and mandible with FRF are the iliac crest, scapula, radial forearm, and fibula flap [5].

Few complications of FRF in the recipient sites are reported such as vascular thrombosis and second primary squamous cell carcinoma (SCC). Many factors have been proposed to be associated with the development of complications after FRF reconstruction such as patients' age, tobacco use, and prolonged surgical time [8–10].

There are four suggested hypotheses for the development of SCC as a complication of FRF; which are the presence of cancer cells into the flap during the implementation of tumor ablation, lymphatic dissemination of the original tumor, the existence of another tumor in the donor site before raising the flap, and the exposure of the skin of the flap to a stimulus in the oral environment that is not normally experienced [8].

The etiology of PG is still unclear. About 30–50% of the patients with PG have a history of local trauma. Infection and poor oral hygiene are frequently reported as triggering factors. Also, the hormonal cause may be added to these factors [4].

In a retrospective study by Jané-salas et al., it is suggested adding incorrect or inadequate prosthesis (implant cap or healing cap, poorly adjusted suprastructures, etc.) as possible causative factors [1]. In general, the association of PG with implants is still controversial.

In the literature, it is reported a presence of hyperplastic/inflammatory response and formation of granulation tissues around implant abutments that are implemented in orally rehabilitated sites by FRF [5]. Anitua and Pinas stated that the implant-related PG seems to be a response to the same stimulus that triggers tooth-related PG. They confirmed the absence of significant correlation between PG and the marginal bone loss around dental implants [6].

In the presented cases, there is only one case of PG around implants in the rehabilitated sites. The poor oral hygiene was the common trigger factor in all the cases, in addition to trauma from the upper left second molar in the first case, pericoronitis related to a partially erupted lower right third molar in the third case, and the poor stability of an upper RPD in the fourth case.

The incidence of recurrence of PG is estimated to be between 2.9 and 8.2%, with a slight increase in cases associated with implants [1]. In the second case, the recurrence was observed probably due to the incomplete elimination of suspected triggering factors. While in the other three cases, the recurrence was not observed. These suggest that the reconstruction by a FRF may be an aggravating condition rather than being a triggering factor of PG.

It seems that the triggering factors are aggravated due to the limitation in oral functions, the difficulty of maintaining the oral hygiene measures following the reconstruction surgery, and the difference in nature between the skin of flap and the normal oral tissues when they are subjected to stimuli, resulting in the development of PG in relation to the site of reconstruction rather than in the common sites that are reported in the literature.

PG is histologically characterized by a prominent capillary growth in hyperplastic granulation tissue. The presence of little vascular fibrotic septa separating a clustered or medullary pattern of the blood vessels leads sometimes to considering PG as a polypoid form of capillary hemangioma [6].

The histological reports of the third and the fourth cases were not with a definitive diagnosis; therefore, these cases were confirmed to be PG through the consultation of an oral pathologist and the clinical picture.

The differential diagnosis of PG includes peripheral giant-cell granuloma, peripheral ossifying fibroma, hemangioma, conventional granulation tissue, and hyperplastic gingival inflammation. In some cases, malignant lesions, such as metastatic carcinoma, melanotic melanoma, or non-Hodgkin's lymphoma, can be a differential diagnosis [3].

## 5. Conclusion

These presented cases suggest that the limitations in oral functions and the difficulty of maintaining the oral hygiene measures due to the FRF reconstruction surgery with the presence of trigger factors such as local trauma, chronic infection, or inadequate prosthesis probably played a role in the development of gingival reactive hyperplastic lesions.
